# Rural vulnerability and institutional dynamics in the context of COVID-19: A scoping review

**DOI:** 10.4102/jamba.v14i1.1227

**Published:** 2022-08-31

**Authors:** Sokfa F. John, Andrew E. Okem, Betty C. Mubangizi, Niyi Adekanla, Londeka P. Ngubane, Ibrahima Barry

**Affiliations:** 1 NRF/SARChI in Sustainable Rural Livelihoods, School of Management, IT and Governance, University of KwaZulu-Natal, Durban, South Africa; 2 School of Life Sciences, College of Agriculture, Engineering and Science, University of KwaZulu-Natal, Durban, South Africa; 3 AIDLINE Research Consult, Lagos, Nigeria; 4 Department of Criminology and Forensic Studies, School of Applied Human Sciences, College of Humanities, University of KwaZulu-Natal, Durban, South Africa; 5 LEMNA (University of Nantes) and ONIRIS, Nantes, France

**Keywords:** vulnerability, rural, resilience, livelihoods, COVID-19, adaptive capacity

## Abstract

This study reviewed the impact of the coronavirus disease 2019 (COVID-19) on pre-existing vulnerabilities in rural communities using the scoping review strategy. It focused on manuscripts published on the topic in 2020. Based on 39 studies that met our inclusion criteria (out of 507 studies), we note that COVID-19 is exacerbating pre-existing rural vulnerabilities, including poverty, remoteness, socio-economic marginalisation and high unemployment. There is limited evidence that rural communities are resilient to the pandemic. Reduction in household expenditures and the community food system are the only reported forms of resilience. Although local institutions are supporting rural communities in responding to the impacts of the pandemic, several institutional dynamics undermine the effectiveness of the response. The increased risk of the pandemic is likely to reduce incomes and standards of living amongst poor communities. Thus, coping strategies were identified such as starting small gardens in communities, diet changes, targeting community markets with produce rather than retailers and food swap using social media, with food swap being the most adopted coping strategy. Although this study does not offer a comprehensive picture of the levels and nature of vulnerability, resilience and institutional dynamics of rural communities in different parts of the world reveal the limitations of existing knowledge of the vulnerability of rural communities in the context of COVID-19. This underscores the importance of further studies on rural vulnerability in the context of COVID-19 that will enable evidence-based responses to the pandemic in rural contexts.

## Introduction

Despite significant progress in the management of infection rates, societies around the world are still struggling to recover from the socio-economic impacts of the coronavirus disease 2019 (COVID-19). Although every level of society has been affected, the intensity of the effect has varied widely across social groups. Rural communities and livelihoods are generally more fragile and are likely to suffer more from shocks and hazards (Jamshed et al. 2020). The COVID-19 pandemic has impoverished the poor and has exacerbated inequality. Informal workers and low-skill workers are severely affected by the lockdown measures (Alzúa & Gosis 2020; Lustig & Tommasi 2021). The poor and the vulnerable are not only affected in terms of lost income but also in terms of how living conditions and future survival are threatened. In this article, we review the impact of the COVID-19 on rural contexts. The article is divided into four sections. The next section examines the concept of vulnerability in socio-ecological systems (SES). The methodology section discusses the methodological approach that underpins the study, followed by the study’s findings. The final section of the article discusses the findings in the context of the broader literature on vulnerability.

## Understanding vulnerability in socio-ecological systems and the socio-economic context

Vulnerability is a complex concept and is variously defined based on discipline and purpose. The Intergovernmental Panel on Climate Change’s (IPCC) (2019:669) definition of vulnerability as ‘the propensity or predisposition to be adversely affected’ is amongst the most often cited. It adds that ‘vulnerability encompasses a variety of concepts and elements, including sensitivity or susceptibility to harm and lack of capacity to cope and adapt’ (IPCC 2019:669).

The literature generally agrees that the term is intimately connected to resilience, sensitivity and adaptive capacity. The meanings of these terms and the precise way they relate to vulnerability are also contested. Most relevant to this study are conceptions of vulnerability in sustainability science and the social sciences. These perspectives overlap and are increasingly shaped by the core concerns of the sustainable livelihoods framework (Moret 2014). They also take a systemic approach that takes seriously the human–biosphere relationship contexts, in addition to the human and biosphere units of vulnerability. For example, Turner et al. (2003) and Turner (2010) advanced the coupled human–environment system (CHES) as a many-sided system that involves processes and connections operating on different spatiotemporal scales and within which vulnerability is located. Gallopin (2006) and Adger (2006) preferred to emphasise social (human) and ecological (biophysical) as the domains of relevance to vulnerability, adaptability and resilience. Gallopin (2006) viewed the socio-ecological system (SES) as one where societal and ecological systems mutually interact and are the natural unit of analysis for research in sustainable development. Adger’s conception of the socioecological system highlights the arbitrariness of treating social and natural systems as distinct units, arguing that both human action and social structures are necessary components of nature. In this usage:

[*N*]atural systems refer to biological and biophysical processes while social systems are made up of rules and institutions that mediate human use of resources as well as systems of knowledge and ethics that interpret natural systems from a human perspective. (Adger 2006:268)

Socioecological systems, as a concept, have evolved to refer to any ‘complex and adaptive’ systems that consist of ‘networks of relations and interactions between humans and nonhuman entities’ (Schlüter et al. 2019). These include systems at different levels and scope – communities, households, institutions and states. This approach enables an analysis of the role that power and institutions play in producing vulnerability and in enabling resilience and coping strategies (Moret 2014).

The contemporary view of the concept of vulnerability places more emphasis on the socio-economic implication of the impact of stressors (Waly, Ayad & Saadallah 2020). From drivers and measurements to coping strategies (Fluharty et al. 2021; Nguyen, Ngo & Tran 2021), socio-economic analysis of the impact of natural disasters has attracted increased interest in the literature. The socio-economic classification scheme could be income, social networks, access to information (collectively tagged as internal factors of socio-economic impact) and factors including national policies, international aid and economic globalisation. Aside from the seemingly generic and exogenous drivers such as income and biophysical factors, specific situations relating to a subject (place or persons) determine the prevailing predictors of vulnerability in such a time and space (Raemaekers & Sowman 2015).

Whilst commonly used as the inverse of vulnerability, resilience appears to be more nuanced. It has been viewed in terms of the ability or extent to which a system preserves its state when confronted with perturbations and stresses. Thus, in the context of SES, resilience is a response (the magnitude of disturbance that can be absorbed whilst remaining in the same state), self-organisation capacity and the capacity to learn and adapt (Turner 2010).

Sensitivity is defined as the ‘extent to which a human or natural system can absorb the impacts without suffering long-term harm or some significant state change’ (Adger & Brown 2009:110). This is closely related to the concept of resilience. However, Adger and Brown (2009) warned that a greater interpretation of the concept of sensitivity is necessary when dealing with ecological and social systems because of the higher level of disagreement regarding what constitutes harm or state change.

Adaptive capacity (capacity of response) is the capacity of a system to cope (Turner et al. 2003) or its capacity of response (Gallopin 2003). It is a component of both vulnerability and resilience (Cohen et al. 2016). The key elements in the use of both capacity of response and adaptive capacity are the ability of a system to adjust to the perturbation or stress it experienced or is experiencing, mitigate potential damage, adjust the system’s sensitivity, increase resilience, minimise exposure, exploit opportunities and cope with the transformation it experiences. Exposure is the ‘nature and degree to which a system experiences environmental or sociopolitical stress’ (Adger & Brown 2009:110). With regard to SES, it is also the duration of a system’s contact with a perturbation or of being subjected to one (Adger 2006). Whether exposure is a component of vulnerability is contested amongst scholars. Adger and Brown (2009) viewed it as an attribute of vulnerability to environmental and social perturbations. However, it appears not to be a necessary quality of vulnerability, because a system can be vulnerable to a perturbation without being exposed to it. But the transformation of a vulnerable system only occurs when there is exposure.

In our study, vulnerability is used within the parameters of the sustainable livelihoods framework (SLF). In the framework, what appears to be clearly articulated is the ‘vulnerability context’ rather than what vulnerability means in such a context. Within the SLF, the UK Department for International Development (DFID), leaning more towards intervention, uses the term ‘vulnerability’ to refer to the conditions of populations and communities that are poorly prepared for disasters and not capable of recovering without external assistance (DFID 1999). At the heart of this is the type of livelihoods of the communities and how these are affected by hazards (Cannon, Twigg & Rowell 2003). Thus, for the DFID, vulnerability and vulnerability analysis must include a predictive element so that proactive interventions can be executed to support relevant institutions and people who are vulnerable (Cannon et al. 2003). Because institutional systems possess an inherently reactive function (Becker 2014; Jia et al. 2020), it is concurred that some approaches to resilience are reactive, given that vulnerable communities and institutions may adapt by addressing the immediate consequences of a hazard.

Institutions are powerful transforming structures within the SLF and an important component of SES. Institutions such as local governments are central to the management of pandemics and disasters. Pre-existing conditions at the institutional level significantly impact the vulnerability or resilience of the whole SES to the adverse impacts of COVID-19, especially in rural settings where institutions are more likely to be weaker and less functional. This limits their ability to swiftly adjust to disasters and take the required measures to efficiently manage the pandemic and minimise its impact on rural communities and livelihoods.

## Methodology

This study seeks to establish the current corpus of knowledge on rural vulnerability in the context of COVID-19. To achieve this aim, a scoping review methodology was implemented (Arksey & O’Malley 2005; Levac, Colquhoun & O’Brien 2010; Munn et al. 2018). Scoping review is gaining traction as an approach to evidence synthesis. Its primary goal is to map available evidence in a particular field or topic based on ‘the volume, nature and characteristics of the primary research’ (Pham et al. 2014). The application of the scoping review strategy is useful when the research topic has not been reviewed extensively (Arksey & O’Malley 2005; Levac et al. 2010). A scoping review is often a prelude to a systematic review (i.e. it enables the researcher to determine the nature, scope and quality of information on a given topic and whether these are sufficient for implementing a systematic review).

The first step in this study was a review of related literature to identify relevant terms for inclusion in our search strategy. Using these terms, we searched the Web of Science (core collection) and Scopus databases. The results from the two databases were downloaded into a spreadsheet and merged into a single document containing 507 studies. These studies were then checked for duplicates and identified, and 41 manuscripts were excluded from the database. After screening the titles of 466 studies, an additional 387 manuscripts were excluded that did not meet our inclusion criteria.

**FIGURE 1:**
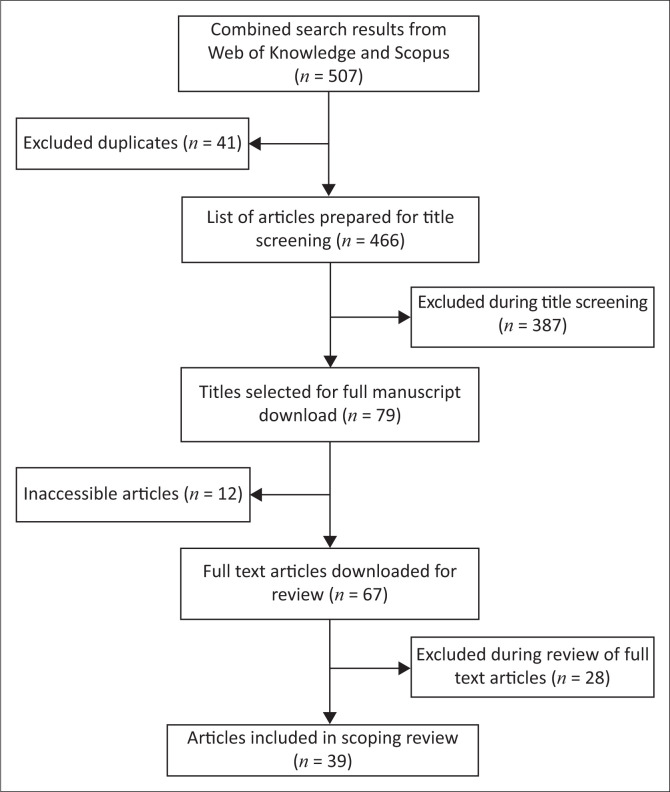
Data screening process.

The next phase entailed downloading the remaining 79 manuscripts for full screening. The full manuscripts for 12 studies could not be accessed; therefore, 67 manuscripts were downloaded. Twenty-eight studies were excluded from our scoping review during the full screening because they did not meet our inclusion criteria. The included articles had to focus on rural contexts, vulnerability and COVID-19, and they had to be nonepidemiological and peer-reviewed. Only 39 manuscripts that met the given inclusion criteria were included in the study. Figure 1 illustrates the data-screening steps used in the study.

Two members of the team reviewed the manuscripts and identified initial themes (including the geographical focus, objectives of the study, key concepts, description of the nature of research, methodology and approach and key results) for data extraction. Based on this initial screening, the team screened the 39 manuscripts and extracted relevant information into a Google Drive document. The next section of this article presents the study’s findings.

### Ethical considerations

This article followed all ethical standards for research without direct contact with human or animal subjects.

## Results

### Characteristics of studies included in the scoping review

The studies included in this review used quantitative and qualitative methods (*n* = 17, respectively). Only four studies used a mixed-method approach, whilst the method used by Ogunkola et al. (2020) was not specified. Of the 39 studies that the authors reviewed, 51% specified the study sample, with a total of 31 421 participants. The average sample size of the studies was 1496 (SD = 1982.33). China accounts for nearly half of the total sample (*n* = 14 611). The minimum sample (*n* = 11) was in Ekoh et al. (2020), whilst the maximum sample (*n* = 8 031) was in Deng et al. (2020). Sample size was not applicable in 16 (41%) studies because of their design (e.g. literature review and analysis of media contents), whilst sample size was not specified for three studies. Two of these studies (Del Brutto et al. 2021; Paganini et al. 2020) used survey design.

The studies were carried out in at least 21 countries across six continents (Table 1). About 33% of the studies (*n* = 13) were from Asia, followed by Africa (28% *n* = 11). Australia and South America had the least number of studies (*n* = 3, respectively).

**TABLE 1:** Location of the studies.†

Regions	Countries	No. of studies
Africa	Sierra Leone (1), Ethiopia (1), South Africa (2), Ghana (1), Zimbabwe (2), Nigeria (2), Uganda (1), Kenya (1) Mozambique (1)	11
North America and Canada	Canada (1), United States of America (5)	6
Europe	Spain (1), Ireland (1), Italy (1)	4
Australia	-	3
Asia	India (4), Pakistan (1), Bangladesh (2), China (5) Cambodia (1) Indonesia (1)	13
South America	Ecuador (1)	1
Not specified	-	1

† , Numbers in parentheses is the total number from each country.

One study each covered Africa, South America and Europe. These were added to the total number of studies for each continent. One study (Paganini et al. 2020) covered four countries: Zimbabwe, Mozambique, South Africa and Indonesia.

### Pre-existing vulnerability conditions

**FIGURE 2:**
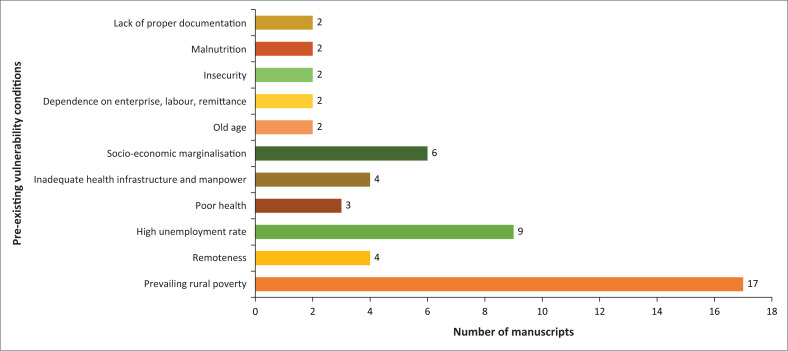
Pre-existing vulnerability conditions that worsened the impact of the COVID-19 pandemic.

Vulnerability was identified in the literature included in this scoping review by considering the exposure or predisposition of rural populations to the adverse effects of COVID-19, sensitivity and stress. Figure 2 presents a summary of the studies that document how pre-existing vulnerability conditions exposed rural settings to the adverse impacts of the COVID-19 pandemic. We identified 11 pre-existing vulnerability conditions across the 39 studies. Prevailing rural poverty was the most identified predisposition to the adverse impacts of COVID-19 and was reported in about 43.5% of studies (*n* = 17). This vulnerability condition was mostly recorded in studies in Asia (*n* = 5) (Ali, Ahmed & Hassan 2020; Alvi & Gupta 2020; Che, Du & Chan 2020; Liu, Pan & Yin 2020), Africa (*n* = 4) (Buonsenso et al. 2020; Dube 2020; Francis & Pegg 2020; Tom & Chipenda 2020) and the USA (*n* = 3) (Haynes-Maslow et al. 2020; Henning-Smith 2020; Peters 2020).

An example of prevailing rural poverty as a vulnerability condition that exposes rural communities to the impacts of the pandemic is offered in Tom and Chipenda’s (2020) article on Zimbabwe. They observe that most families in Zimbabwe were already vulnerable to poverty and other economic shocks, and the programmes that are supposed to protect them are weak and barely benefitted them. They also observed that Zimbabwe’s rural poverty was at 76.9%, having increased by 31% since 2012. The impact of the pandemic will likely increase the proportion of the poor in rural Zimbabwe. Findings of the study by Luo et al. (2020) are similar to Tom and Chipenda (2020). According to Luo et al. (2020), there is a high likelihood that rural households in China which had come out of poverty recently would fall back into poverty because of the pandemic. They noticed that the pre-existing conditions of rural households made them a vulnerable population which is predisposed to the adverse impacts of COVID-19.

Higher rates of unemployment in rural areas were reported in 23% (*n* = 9) of the studies as an exposure factor. Three of these were in Africa (Buonsenso et al. 2020; Janssens et al. 2021; Tom & Chipenda 2020) and three in Asia (Ali et al. 2020; Che et al. 2020; Liu et al. 2020). No study mentioned unemployment as an exposure factor in South America, whilst one study each identified unemployment as an exposure in North America (Peters 2020), Australia (Kent et al. 2020) and Europe (De Luca, Tondelli & Åberg 2020). Socio-economic marginalisation was reported in six studies (Alvi & Gupta 2020; Dube 2020; Henning-Smith 2020; Haynes-Maslow et al. 2020; Peters 2020; Surendra 2020) and remoteness of rural areas in four studies (Ali et al. 2020; Haynes-Maslow et al. 2020; Henning-Smith 2020; Meredith et al. 2020). Malnutrition (Cattivelli & Rusciano 2020; Francis & Pegg 2020) and insecurity (Cattivelli & Rusciano 2020; Francis & Pegg 2020) were reported in two studies each. Other least-reported exposures were poor health (Henning-Smith 2020; Jones et al. 2020; Surendra 2020), poor documentation (Meyer et al. 2021; Peters 2020), dependence on enterprise, labour and remittance (Janssens et al. 2021; Mahmud & Riley 2021), old age (Ekoh et al. 2020; Henning-Smith 2020) and inadequate health infrastructure and manpower (Surendra 2020; Tom & Chipenda 2020).

The total exposure or predisposition to the adverse effects of COVID-19 was highest in studies in Africa. Out of the 11 predisposition conditions observed, only remoteness was not identified in Africa. This is followed by North America with eight conditions, whilst Asia and Europe each had five conditions. Only three were reported in Australia (prevailing rural poverty, high unemployment rate, poor health).

**FIGURE 3:**
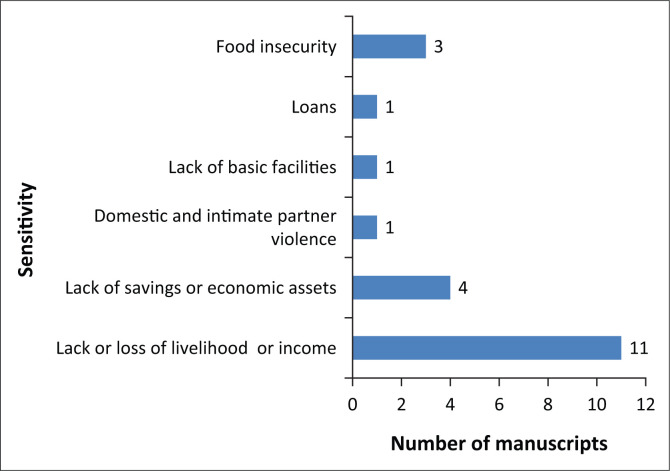
Sensitivity of rural communities to the impact of COVID-19.

Figure 3 identifies the sensitivity of rural communities to the impact of COVID-19. The factors identified here make it difficult for communities to absorb the impact of COVID-19 without long-term harm or a significant change in the state of their livelihoods (Adger & Brown 2009:110). Thus, these are factors that increase sensitivity to the adverse impact of COVID-19. The lack or loss of livelihood and income was the most cited sensitivity factor in rural communities. This was reported in 28% (*n* = 11) of the studies, followed by a lack of savings or economic assets (*n* = 4). Four of the studies that cited the absence of livelihoods or income are from Asia (Hamadani et al. 2020; Liu et al. 2020; Surendra 2020), three from Africa (Dube 2020; Mahmud & Riley 2021; Meyer et al. 2020), two from North America (Henning-Smith 2020; Peters 2020) and only one each from Australia (Kent et al. 2020) and Europe (De Luca et al. 2020).

Meyer et al. (2020), for example, found the changes in employment status amongst female garment works in Ethiopia to be high, as 41% of their respondents had either lost their jobs or were put on leave at the time of their study. Surendra (2020) observed several sensitivity factors affecting rural communities in India in the context of COVID-19, coded in Figure 3 as subcategories of lack of basic facilities, food insecurity and lack of savings and economic assets. Some of these include inadequate access to water and sanitation, high poverty rates, over-dependence on natural resources for livelihoods, lack of clean energy for cooking and lack of access to medical facilities and other amenities.

Lack of savings was only reported in North America and Asia (Cattivelli & Rusciano 2020; Peters 2020). Loans (Janssens et al. 2021), lack of basic facilities (Surendra 2020) and domestic and intimate partner violence (Hamadani et al. 2020) were reported in one study each whilst food insecurity was reported in three studies (Cattivelli & Rusciano 2020; Hamadani et al. 2020; Meyer et al. 2021). Most respondents in Meyer et al. (2021), for example, were food insecure and said they were worried about not having enough food. The study that reported intimate partner violence (Hamadani et al. 2020) showed that although incidences of partner violence existed before COVID-19, according to most of the 2174 women studied, it increased during lockdown in rural Bangladesh. Forms of violence included emotional, physical and sexual violence.

Asia had the most reported cases of sensitivity, as all conditions, except loans, were reported in at least one study on a location in Asia. Three sensitivity conditions (lack or loss of livelihoods, loans and food insecurity) were applicable to African locations (Janssens et al. 2021; Mahmud & Riley 2021; Meyer et al. 2021). Only two conditions each applied to Europe (food insecurity and lack or loss of livelihoods) and North America (lack or loss of livelihoods and food insecurity). The least sensitive location was Australia, where only the lack or loss of livelihood or income was reported (Kent et al. 2020).

**FIGURE 4:**
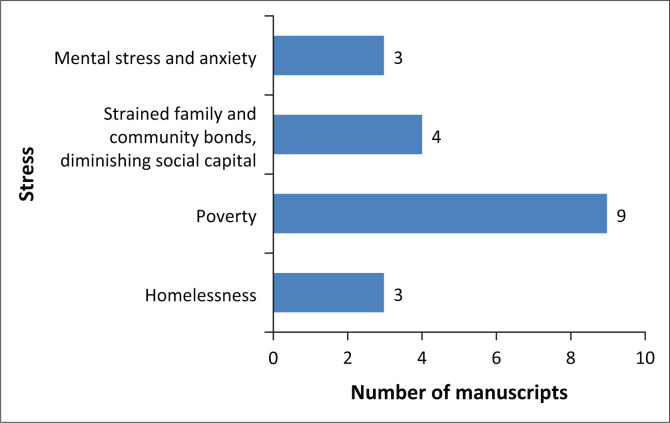
Stress factors associated with COVID-19.

The restrictions that come with the COVID-19 control measures – such as lockdown, social distancing and wearing of face masks – are unprecedented in recent human history. In the studies included in this review, four stresses (mental stress and anxiety, strained relationships, poverty and homelessness) associated with the pandemic were identified (Figure 4). Stress, as defined by Gallopin (2003), refers to an increase in pressure, usually from within a system. Stress here denotes factors that increased pressure on vulnerable populations during the pandemic. Some of these may also have been factors that not only predisposed them to the adverse impacts of the pandemic, but also functioned as stresses during the pandemic. As Figure 4 shows, poverty was the most identified stress across the studies (*n* = 9), whilst mental stress, anxiety and homelessness were the least (*n* = 3). However, of note is that the most identified stressor (poverty) was cited in only 23% of the studies. Unlike in other studies, poverty as a stress in Cattivelli and Rusciano (2020) is presented in terms of the high risk of relative poverty in Naples, Italy, considering that its poverty risk level is the highest in the European Union.

All four stresses were identified in Asia (Alvi & Gupta 2020; Luo et al. 2020). Only homelessness did not appear in any studies on Africa, whilst North America and Australia both reported only homelessness and poverty (Schiff et al. 2020; Usher et al. 2021). Schiff et al. (2020) argued that rural homelessness in Canada, whilst often ignored, increases the vulnerability of the rural population to the COVID-19 pandemic.

### Resilience to the COVID-19 pandemic

Rural communities are faced with various pre-existing vulnerability conditions and have, over the years, developed resilience in response to these vulnerability factors. To identify indications of resilience during the COVID-19 pandemic in the studies reviewed, the following three aspects were considered: resistance or the capacity to remain or return to normal in response to COVID-19, capacity to adapt and coping strategies.

**FIGURE 5:**
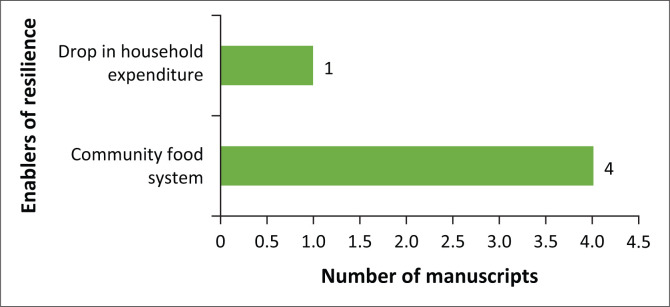
Enablers of resilience to the impact of COVID-19.

In this study, only reduction in household expenditure and community food systems were identified as enabling resilience to the impact of COVID-19 on livelihoods in rural contexts (Figure 5). Two studies (Janssens et al. 2021; Paganini et al. 2020) highlighted community systems in terms of enhancing food security whilst Cattivelli and Rusciano (2020) and Haynes-Maslow et al (2020) identified community food systems in Europe and North America. Only one study (Janssens et al. 2020) identified a drop in household expenditure as a resilience factor.

**FIGURE 6:**
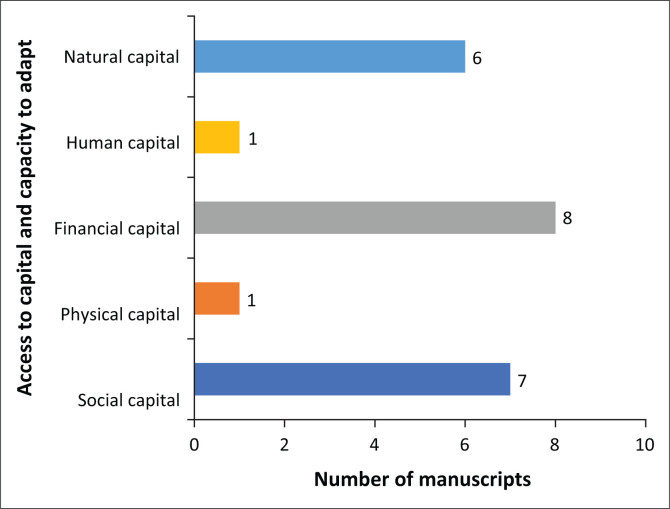
Access to capital and capacity to adapt.

The capacity to adapt to shocks is an important characteristic of a resilient community. The adaptive capacity across the 39 studies were identified using the five sustainable livelihood capitals (Figure 6). Financial and social capital were the most cited across the studies (*n* = 8 and 7, respectively), followed by natural capital (*n* = 6), whilst human and physical capital were the least cited (*n* = 1, respectively).

Social and natural capital were identified in studies on Africa (Janssens et al. 2021; Mahmud & Riley 2021; Meyer et al. 2021; Paganini et al. 2020), Europe (Cattivelli & Rusciano 2020; De Luca et al. 2020), and Asia (Che et al. 2020; Liu et al. 2020; Surendra 2020). Financial capital was only identified in studies from Africa (Dube 2020; Janssens et al. 2021) and Asia (Ali et al. 2020; Che et al. 2020). Only studies from Europe reported both human and physical capital (Cattivelli & Rusciano 2020; De Luca et al. 2020) whilst no study from North America and Australia reported on any of the five capitals.

**FIGURE 7:**
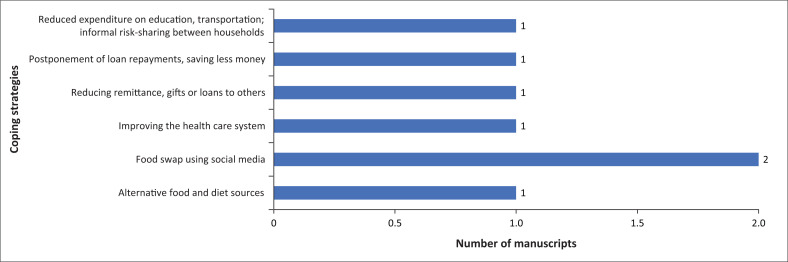
Coping strategies during the pandemic.

A total of six coping strategies were identified but these were reported in only a few. Food swap using social media was identified as the most adopted coping strategy in only two studies: one from Africa (Paganini et al. 2020) and the other in Europe (Cattivelli & Rusciano 2020). Figure 7 shows that each of the other coping strategies was identified in only one study, respectively, and they are all from three studies on African rural locations (Janssens et al. 2021; Kwegyir Tsiboe 2020; Paganini et al. 2020).

Paganini et al. (2020) observed that farmers in southern African countries and Indonesia demonstrated a capacity to adapt to the COVID-19 pandemic by starting small gardens in their communities, changing their diets and targeting community markets with their produce rather than retailers. In Toraja (Indonesia), for example, nearly half of the farmers, 61% of whom were women, implemented their food security solutions to cope with the pandemic. They started vegetable gardens, spent less and changed their diets. Over 90% of these farmers achieved their coping goals through co-operation with either family or neighbours (Paganini et al. 2020). Whilst the coping strategies generally indicate a reduction in spending and extension of resources to others, Hamadani (2020) showed that families in rural Bangladesh also combined their savings with procuring loans and accessing relief from other sources, including the government.

### Institutional dynamics in responding to the COVID-19 pandemic

**FIGURE 8:**
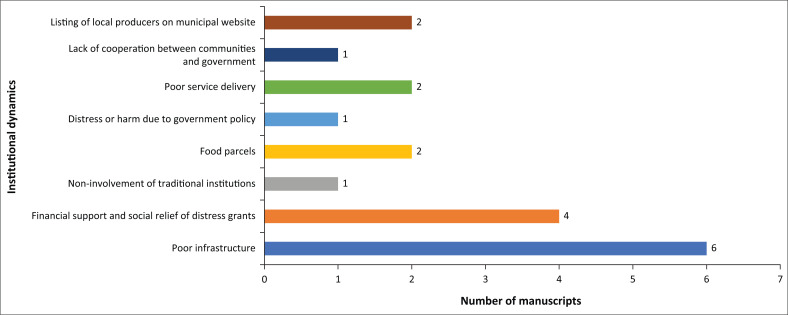
Institutional dynamics in responding to the COVID-19 pandemic.

Figure 8 presents the institutional dynamics concerning COVID-19 in rural contexts. It shows several institutional support or response measures and barriers to institutional response in helping residents of rural communities to cope with the impact of the pandemic. Three forms of institutional support were identified: listing of local producers on municipal websites (*n* = 2) (Europe), food parcels (*n* = 4) (Europe and the USA) and financial support and distress grants (*n* = 4) (Africa, Europe and Asia) (Kim et al. 2020).

De Luca et al. (2020) described how institutional websites, complemented by other digital platforms, helped rural dwellers during the pandemic. Municipalities listed local producers’ food and other goods on their websites to enable community members to buy these products directly from producers. These producers also used their websites, phones, network of local farmers and Facebook pages, amongst other things, to sustain their livelihoods during the pandemic. Whilst food and financial support from the government were acknowledged, there are indications that these were insufficient or inadequately administered. For example, Luo et al. (2020) observed that whilst the government provided social security such as staple foods and cash for poorer families for 8 million (including older people, widows and lactating mothers) only 12% of their sample of 1733 benefitted from this support.

Poor infrastructure was the most cited (*n* = 6) institutional factor affecting the COVID-19 response, as observed in studies from all regions except Australia. This is followed by poor service delivery (*n* = 2) observed in Asia (Surendra 2020). Other issues such as the lack of co-operation between communities and government (Ali et al. 2020) and distress or harm because of government policy and the noninvolvement of traditional institutions (Che et al. 2020) were identified in studies from Asia.

## Recommendations for improving responses to the COVID-19 pandemic

**FIGURE 9:**
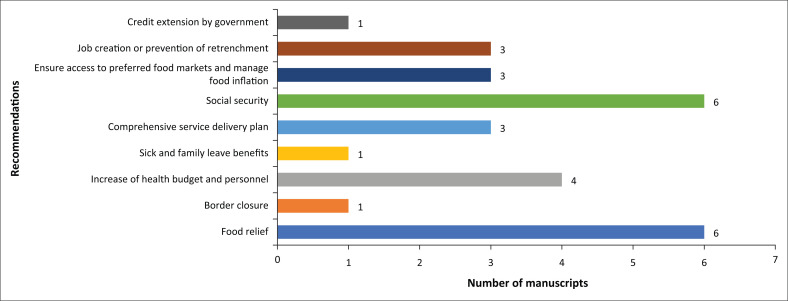
Recommendations for improving the response to COVID-19.

Figure 9 presents nine recommendations for responding to the COVID-19 pandemic across the studies, with social security and food relief being the most cited recommendations (*n* = 6, respectively). An increase in health budget and personnel was cited in only four studies. About half of these recommendations were from studies in Africa (Ekoh et al. 2020; Paganini et al. 2020; Kwegyir Tsiboe 2020), whilst the remainder are spread across studies from the other continents.

Ekoh et al. (2020), for example, recommended that social workers advocate for more interest from government and NGOs in the welfare of older people in rural areas. They observed that this population should be provided with relief materials and their dependence on their informal support network for financial and material support should be addressed with a long-term policy and social security solutions. Salzwedel et al. (2020), writing about farmworker childcare issues in the context of COVID-19 in the USA, recommended federal aid packages such as benefits for family or sick leave and up to 12 weeks of paid time off.

## Discussion

The literature in this scoping review approached the vulnerability of rural communities in the context of COVID-19, using diverse methods and techniques. This indicates multiple interests and perspectives on rural livelihoods and vulnerability. Viewed together, the literature offers a relatively dynamic picture of the vulnerability of rural communities in the context of COVID-19. The reliance of 41% of the studies on literature, media contents, reports and other documents rather than empirical studies could reflect the challenges posed by the COVID-19 pandemic and related restrictions on accessing populations for research.

Most of the relevant literature on rural vulnerability in the context of COVID-19 is about Asian and African contexts (33.3% and 28.2%, respectively). These two contexts make up 61.5% of the studies, whilst there was only one study from South America. It follows that the vulnerability identified in the study was higher, overall, in Asia and Africa than in any other region. In other words, these societies and their livelihoods either experienced or were likely to experience higher degrees of harm and other hazards (Turner et al. 2003) such as COVID-19. Whilst this is a factor of the level of vulnerability corresponding with the literature in this scoping review, it is consistent with broader literature, which shows that rural communities in Asia and Africa are more fragile and vulnerable to shocks than those in Europe, Australia and North America, as the shock could culminate in a disastrous social and economic emergency (OECD 2020).

Our review found a generally high vulnerability and low resilience of rural communities concerning the adverse impacts of the COVID-19 pandemic. In terms of the specific markers of vulnerability, the preconditions that mostly exposed communities to the adverse impact of the pandemic were prevailing rural poverty and high rates of unemployment, not only concerning Africa, but also significantly the case in other regions. Poverty appears to be the central vulnerability condition identified in this study under each indicator. Poor people are more vulnerable to shocks, regardless of their origin. Pre-existing poverty implies any impact on their asset or consumption level that threatens subsistence and long-term prospects, and they have fewer resources to reduce risks or to cope with the shock when it occurs. The rural poor in middle-income and low-income countries are particularly at risk because of the depth of their poverty, high population density, reduced remittances and the limited capacity of the state to respond. From the SES perspective of Zimmerman, Willig and Hernández-Delgado (2020), these factors, especially poverty, are the ‘points of weakness’ which negatively impact the dynamics and status of the SES as a whole because of exposure to the COVID-19 pandemic.

The low resilience observed in this scoping review was because of the low levels of resistance, coping strategies and adaptive capacity that the studies indicated. Only 5 out of the 39 studies, for example, highlighted some level of resilience to the adverse impact of the COVID-19 pandemic on rural livelihoods. This was primarily concerning efforts of local communities to ensure food availability (*n* = 4 studies). It is highly unlikely that these rural communities will return to normal as a response to the shock of the pandemic. Adaptive capacity was especially low for the most vulnerable contexts, and the most identified coping strategy (food swap) was only reported in two studies. Whilst this could be highlighting the need for more studies on the resilience of rural communities to the pandemic, it could also indicate that the resilience of rural communities to disasters and shocks is very low. The latter is supported by Hallegatte et al. (2020), who argued that poor people are more vulnerable to shocks – regardless of their origin. Any impact on poor people’s assets or consumption level threatens their subsistence and long-term prospects. This is because they have fewer resources to reduce risks or to cope with shocks. After a shock, when income and wealth are reduced and people’s health is affected, broad safety net programmes may automatically scale up if they are designed to respond to changes in household situations.

Government and other institutions made significant efforts to control the spread of COVID-19 and manage its impact on livelihoods. The authors found both positive (*n* = 11) and negative (*n* = 8) assessments of such efforts in the studies that they reviewed. Although difference in terms of the number of studies was small, it is significant considering that the dominant inclination of the literature was to argue that institutional efforts were limited and often inadequate.

With regard to livelihoods, the inadequacies of institutions appear to be mainly linked to the lockdown period. Buonsenso et al. (2020) observed that African countries responded adequately to COVID-19 concerning controlling infection rates by instituting timely lockdowns, quarantines and diagnostic centres. However, they argue that in the rural villages that they studied (mostly dependent on fishing, tourism and minor markets and where residents lost over 51% of their income during the lockdown) and others around Africa, governments did not offer any support. Much of institutional inability to adequately respond can be attributed to poor infrastructure (*n* = 11) and weak governance arrangements expressed in the form of lack of co-operation between communities and government, particularly the noninvolvement of traditional institutions. The literature suggests not only a range of institutional response measures, but also highlights barriers to institutional response in helping rural communities to cope with the impacts of the pandemic. The institutional dynamics observed in this study highlight the critical role of institutions as transforming structures and processes of SES in the context of livelihoods (DFID 1999). Institutions can create or increase resilience or vulnerabilities and alleviate or worsen livelihood conditions by how they respond to hazards and the general well-being of the population. Pre-existing institutional conditions act as barriers or enablers of COVID-19 prevention, management and response. They relate to the capabilities and stock of resources an institution has at its disposal and the type of networks a given institution can draw upon for resource support.

The different themes that indicated vulnerability, levels of resilience and institutional dynamics cut across different spheres of the natural and social world. Our study is therefore another example of vulnerability in a coupled human–environment system (CHES) (Turner 2010) or socio-ecological system (SES) (Adger 2006; Gallopín 2006). Both involve processes and relationships operating in the connected human (social) and biophysical (ecological) spheres as the site of vulnerability and resilience. Rural communities are critical to this complex and adaptive system, and the high levels of vulnerabilities in rural locations of India and many African countries confirm the complex and intertwining challenges to the SESs of these locations.

## Conclusion

This study adopted the scoping review approach to examine rural vulnerability in the context of the COVID-19 pandemic. Based on 39 studies that met our inclusion criteria, it was found that COVID-19 is exacerbating pre-existing vulnerability conditions (including poverty and remoteness) in rural communities. The studies included in the review reveal that rural contexts have low resilience to shocks and are therefore likely to experience long-lasting impacts of shocks because of their limited adaptive capacity. Furthermore, levels of vulnerability, resilience and capacity to respond are differentiated by geographical contexts. Africa and Asia are the most vulnerable, the least resilient and the regions with the least capacity to respond.

Although the studies provided evidence that institutions in rural contexts are responding to the pandemic, these were either largely absent in most of the contexts examined or inadequate in terms of helping rural communities to cope with the impacts of the pandemic.

Whilst this study does not offer a comprehensive picture of the levels and nature of vulnerability, resilience and institutional dynamics of rural communities in different parts of the world, it reveals the limitations of existing knowledge on the vulnerability of rural communities and institutions in the context of COVID-19. This underscores the importance of further studies on rural vulnerability and institutional dynamics in the context of COVID-19. Such studies will enable evidence-based responses to the pandemic in rural contexts.
